# Cold Feet While Traveling

**Published:** 1866-12

**Authors:** 


### COLD FEET WHILE TRAVELING,

If boots are treated as above, and just before going out of doors the stockings are removed, and both feet and stockings are well dried before the fire, the feet will feel comfortably warm for several hours; it is the moisture or steam about the feet which often makes them feel cold by the out-door air condensing them. No one should travel in winter with tight-fitting shoes; they arrest the circulation: this induces coldness, causing a general feeling of discomfort all over the body, even making the mind fretful and irritable. A woolen stocking will alone keep the feet warmer than the same stockings and a pair of tight boots besides. If a person has a good circulation, the feet will get warm of themselves if the tight boots are removed. No one can go to bed with cold feet without doing themselves a positive injury; and it is always best in winter-time, even if the feet do not feel cold, at bed-time to draw off the stockings and hold the feet to the fire or stove, rubbing them meanwhile with the hand, until they are perfectly dry and comfortably warm in every part; it is a pleasant operation of itself, and ought not to be dispensed with for a single night from October to May; it is one of the best anodynes; it allows a person to fall asleep in five minutes, who, with cold feet, would have remained awake for half an hour or more, and even then the sleep will be unrefreshing and dreamy.

